# Next day sentinel node biopsy for melanoma after lymphoscintigraphy using ^99m^Tc-labelled nanocolloid does not adversely affect long-term outcomes

**DOI:** 10.1007/s12149-024-01980-y

**Published:** 2024-09-16

**Authors:** Amit Roshan, Terouz Pasha, Georgios Kounidas, Suzanne Murphy, Luigi Aloj, John Buscombe, Animesh Patel, Amer Durrani

**Affiliations:** 1 Department of Plastic and Reconstructive Surgery, Cambridge University Hospitals NHS Foundation Trust, Cambridge, UK; 2 Department of Nuclear Medicine, Cambridge University Hospitals NHS Foundation Trust, Cambridge, UK; 3 Department of Radiology, University of Cambridge, Cambridge, UK; 4 Cancer Research UK Cambridge Institute, University of Cambridge, Robinsons Way, Cambridge, CB2 0RE UK; 5 Present Address: Department of Plastic Surgery, Royal Free London NHS Foundation Trust, London, UK; 6 Present Address: Department of Plastic Surgery, Mid and South, Essex Hospitals NHS Foundation Trust, Chelmsford, UK

**Keywords:** Melanoma, Sentinel lymph node, Timing, Nanocolloid

## Abstract

**Objective:**

Sentinel Lymph Node Biopsy (SLNB) is an important management tool for early-stage melanoma. Different radiopharmaceuticals are used internationally to localise the sentinel node using lymphoscintigraphy (LSG) before surgery. Recent reports have suggested that a delayed interval between LSG and SLNB using ^99m^Tc-labelled nanocolloid tracer has an adverse survival impact, but not with ^99m^Tc-labelled antimony sulphide colloid. This study aims to analyse survival outcome in a prospective cohort of melanoma patients undergoing same day or next day SLNB after LSG using ^99m^Tc-labelled nanocolloid.

**Methods:**

Outcome data were reviewed for patients undergoing SLNB, stratified by time interval between LSG and SLNB at a single UK academic centre. Kaplan–Meier survival analysis was used to assess overall survival (OS), melanoma-specific survival (MSS) and progression-free survival (PFS). Cox multivariable regression analysis identified independent risk factors.

**Results:**

925 patients had LSG using the ^99m^Tc-nanocolloid tracer between 2009 and 2019, with a median follow-up of 6.36 years. No difference was seen on univariate analysis in OS, MSS, PFS, or nodal recurrence between patients undergoing same day or next day SLNB (Log-rank *P* = 0.437, 0.293, 0.587, 0.342 respectively). In addition, nodal recurrence as first site or anytime site of recurrence in SLNB negative patients was similar between the groups (Log-rank *P* = 0.093 and 0.457 respectively). Stratified analysis of time did not demonstrate an outcome difference (MSS Log-rank *P* = 0.938). Cox multivariable regression did not show time interval to independently influence OS, MSS or PFS.

**Conclusions:**

We do not find a significant effect on long-term outcomes when SLNB is performed the day after LSG with ^99m^Tc-labelled nanocolloid tracer. We infer that tracer migration is not clinically significant within 24 h of injection based on long term clinical outcome data.

**Supplementary Information:**

The online version contains supplementary material available at 10.1007/s12149-024-01980-y.

## Introduction

Sentinel lymph node biopsy (SLNB) stages the regional lymph nodes for patients presenting with primary cutaneous melanoma clinically localised to the skin. The Multicenter Selective Lymphadenectomy Trial (MSLT-I) demonstrated the accuracy, and low morbidity associated with this approach making it widely accepted as the standard of care for early melanoma management [1]. Although this trial did not show a statistically significant overall survival benefit for patients undergoing SLNB, latent subgroup analysis showed that patients with intermediate thickness melanomas had improved outcomes when undergoing SLNB in conjunction with immediate completion lymphadenectomy (CLND) [1]. It has thus been validated as a valuable staging and prognostic tool, with potential outcome benefits in some groups.

Central to the utility of SLNB is the diagnostic accuracy of the procedure itself. Considerable effort has been placed in understanding the pitfalls of the procedure, and improving elements that potentially lead to false results [2]. The identification of the sentinel lymph node is done via a triple localisation technique involving nuclear medicine lymphatic imaging with an injected radiotracer prior to surgery, a second blue contrast dye injection immediately intraoperatively to provide visual cue to the surgeon, and a hand-held gamma probe during surgery. Most European centres use the ^99m^Tc-labelled nanocolloid radiotracer, although ^99m^Tc-labelled Antimony Sulphide colloid is prevalent in Australasia, and ^99m^Tc-labelled Sulphur colloid is widely used in North America. The European Association of Nuclear Medicine practice guidelines recommend using the ^99m^Tc-labelled nanocolloid for surgery scheduled either on the same day, or on the next day with similar localisation characteristics in both schedules [3].

Two recent reports have suggested the using the ^99m^Tc-labelled nanocolloid for SLN identification can have an adverse impact on outcomes for melanoma if used in a next day surgery protocol [4, 5], but no such effect is seen in a separate report using the ^99m^Tc-labelled antimony sulphide colloid [6]. In this study, we aimed to review the long-term outcomes from ^99m^Tc-labelled nanocolloid to localise the SLN in melanoma on the same day or next day surgery in an independent cohort. A long-term follow-up was critical to minimise the risk of false negative results.

## Materials and methods

### Patients

This study included patients Stage I-IIC primary cutaneous melanoma according to the eighth edition of the American Joint Committee on Cancer classification [7] at the Cambridge University Hospitals NHS Trust, United Kingdom. All patients who had lymphoscintigraphy using the ^99m^Tc-labelled nanocolloid between December 2009 and December 2019 were included from a prospectively collected database. Patients who had multiple melanomas, and any patients who subsequently did not undergo surgery for either medical reasons or drainage identified to multiple nodal basins (> 4) were excluded.

Institutional Ethics review was granted under the Cambridge University Hospitals Electronic Health Records Research and Innovation Database (ERIN, A096904).

The STROBE guidelines for reporting observational cohort studies were followed.

### Sentinel node localisation and identification

Intradermal injections of up to 20 MBq ^99m^Tc-labelled nanocolloid (GE Healthcare, Amersham, UK) were administered adjacent to the primary melanoma site. Early dynamic imaging, and delayed planar imaging was undertaken to characterise the lymphatic drainage and identify the sentinel lymph nodes. Lymphatic collectors and their draining sentinel nodes were identified and marked on the skin surface to aid intraoperative localisation. Single photon emission computed tomography with CT (SPECT-CT) was additionally used if the draining lymph nodes were in the neck for improved anatomical localisation [8]. Studies were acquired on a GE Healthcare Discovery NM/CT 670 dual headed gamma camera SPECT CT system. Dynamic planar views of the region of interest were initially obtained for up to 10 min (60 s per frame) following administration of the tracer using a Low Energy High Resolution (LEHR) collimator. When required, SPECT CT views were obtained (20 s per view, step and shoot mode, 60 views per camera covering 360 degrees, LEHR collimator). CT was carried out with a low dose protocol full helical, 120 kV, 120 mA and reconstructed at 2.5 mm slice thickness. Planar views and reconstructed tomographic images (CT, SPECT and fused) were available to review on the Trust PACS.

Two groups of patients were identified depending on the timing of the surgery in relation to the lymphoscintigraphy. On the same day surgery group, patients had tracer injections typically between 08:00 and 11:00, and surgery in the afternoon. From November 2012, some patients began a next day surgery protocol to accommodate rising demand and manage scheduling workload. In this group, tracer injections were typically between 12:00 and 15:30, and surgery was performed the next day. With the publication of two reports suggesting a poorer survival outcome with next day surgery, we discontinued the next day surgery protocol in Feb 2020 [4, 5].

At surgery, Patent Blue^®^ V dye (Guerbet, Villepinte, France) was injected immediately adjacent to the diagnostic excision scar. Intraoperative localisation of the sentinel lymph nodes was with a dual technique, using a hand-held gamma probe and visualisation of blue staining. Radioactivity was recorded, and the sentinel node was preserved in formalin. Histological examination was undertaken following the EORTC protocol with haematoxylin and eosin stains, and aided by immunohistochemistry [9].

### Follow up and identification of disease recurrence

Patients with no microscopic melanoma identified in the sentinel lymph node(s) or wide local excision specimen, were followed up in dedicated skin cancer outpatient clinics at 3 monthly intervals in the first 3 years, and 6 monthly for an additional two years as per UK guidelines [10, 11].

Patients with microscopic melanoma had baseline whole-body imaging, and were offered either completion lymph node dissection (CLND), observation or Ultrasound-guided lymph node basin imaging according to the Multicenter Selective Lymphadenectomy Trial (MSLT-II) [12]. In 2019, adjuvant systemic therapy was approved in the UK, and 15 patients had either adjuvant biological or immune therapy.

Any suspicion of metastatic disease was investigated with appropriate imaging and tissue biopsy was sought where appropriate.

### Statistical analysis

The same day surgery group was defined as any patient undergoing LSG and SLNB on the same calendar day, with any patient having LSG and SLNB on consecutive days in the next day surgery group. In the time stratified subgroup analysis, 4 groups of patients were identified: same day surgery with LSG to SLNB interval < 6 h, same day surgery with LSG to SLNB ≥ 6 h, next day surgery with LSG to SLNB interval < 24 h, and next day surgery with LSG to SLNB interval ≥ 24 h.

Analysis was using Graphpad Prism^®^ version 10.1.1 (GraphPad Software, San Diego, California, USA). Patient characteristics and histopathological features in each group were summarised with descriptive statistics. Tests included Chi-square test to compare categorical variables, *t* test to compare continuous variables, and Kaplan–Meier survival analysis to compare overall survival (OS), melanoma-specific survival (MSS), progression-free survival (PFS), nodal recurrence and distant progression. Survival differences between cohorts were evaluated with the log rank test. Cox multivariable proportional hazards analysis was used to assess the independent impact of surgery timing on survival, with adjustment for potential confounders. Statistical tests were two-sided, and *P* < 0.05 was considered statistically significant.

False negative rate (FNR) was defined as the ratio between false negatives and all patients with anytime lymph nodal involvement (the sum of false negative and true positives).

## Results

925 patients had preoperative lymphoscintigraphy with ^99m^Tc-labelled nanocolloid radiotracer. Of these, 696 had LSG and SLNB on the same day, and 229 patients had these on consecutive days. Median follow up was 6.36 years in the overall cohort (6.54 years same day cohort, and 6.11 years next day cohort, Table 1).

**Table 1:** Demographic and tumour characteristics in patients according to timing of surgery after lymphoscintigraphy

	Same day surgery (*n* = 696)	Next day surgery (*n* = 229)	*P*^‡^
Follow up period (years)			
Median (IQR)	6.54 (4.06–9.24)	6.11 (4.27–8.06)	0.009^§^
Age at primary diagnosis (years)			
Median (IQR)	59.0 (47.9–68.4)	61.7 (50.1–70.0)	0.023^§^
Gender			
Male	351 (50.4)	119 (52.0)	0.687
Breslow thickness (mm)^a^			
Median (IQR)	1.6 (1.1–2.7)	1.6 (1.1–2.6)	0.623^§^
Breslow thickness (mm)^a^			
≤ 1.0	142 (20.5)	56 (24.6)	0.490
1.1–2.0	298 (43.1)	88 (38.6)	
2.1–4.0	173 (25.0)	55 (24.1)	
> 4.0	79 (11.4)	29 (12.7)	
Ulceration^b^			
Yes	123 (18.3)	58 (26.2)	0.011
No	548 (81.7)	163 (73.8)	
Tumour site			
Limb	389 (56.0)	120 (52.4)	0.522
Trunk	223 (32.1)	76 (33.2)	
Head or neck	83 (11.9)	33 (14.4)	

Values are percentages unless otherwise indicated

^a^Data available for 692 patients on the same day surgery group and 228 patients in next day surgery group

^b^Data available for 671 patients on the same day surgery group and 221 patients in next day surgery group

^‡^*χ*^2^ test

^§^Unpaired *t* test

### Patient, tumour and lymph node characteristics

Demographic and tumour characteristics stratified to the same day or next day SLNB groups are detailed in Table 1. The groups had no significant differences between the two groups on key predictors of outcome including Breslow thickness, Gender or tumour location. The next day surgery cohort did have a higher proportion of ulcerated primary tumours (26.2% vs. 18.3%, *P* = 0.011) and a higher median age (61.7 years vs. 59.0 years, *P* = 0.023).

Key indicators relating to the lymph nodes and follow up are summarised in Table 2. There was no observed difference in the sentinel node status, number of sentinel nodes removed at surgery, number of positive sentinel nodes, uptake of CLND, nonsentinel node involvement at CLND, adjuvant systemic therapy, site of first relapse, lymph node relapse (either as first site or at any time during follow up). The False negative rates were 14.9% in the same day surgery group and 15.6% in the next day surgery group (*P* = 0.915).

**Table 2:** Nodal and follow up characteristics in patients according to timing of surgery after lymphoscintigraphy

	Same day surgery (*n* = 696)	Next day surgery (*n* = 229)	*P*^‡^
Sentinel node^a^			
Negative	539 (77.8)	191 (83.4)	0.069
Positive	154 (22.2)	38 (16.6)	
Number of sentinel lymph nodes excised^b^			
Median (IQR)	1.0 (1.0–2.0)	1.0 (1.0–2.0)	0.556
Number of sentinel lymph nodes excised^b^			
0	10 (1.4)	4 (1.8)	0.652
1	339 (48.9)	120 (52.6)	
2	213 (30.7)	58 (25.4)	
3	76 (11.0)	30 (13.2)	
4	31 (4.5)	8 (3.5)	
≥ 5	24 (3.5)	8 (3.5)	
Number of positive sentinel lymph nodes^c^			
0	540 (77.8)	190 (83.0)	0.179
1	128 (18.4)	31 (13.5)	
2	23 (3.3)	6 (2.6)	
3	3 (0.4)	1 (0.4)	
4	0	1 (0.4)	
CLND			
Performed	103 (67.8)	24 (63.2)	0.590
Not performed	49 (32.2)	14 (36.8)	
Nonsentinel nodes in CLND			
Negative	85 (82.5)	17 (77.3)	0.564
Positive	18 (17.5)	5 (22.7)	
Adjuvant systemic therapy			
Yes	10 (1.4)	5 (2.2)	0.438
No	686 (98.6)	224 (97.8)	
Site of furthest first relapse			
No relapse	542 (77.9)	184 (80.3)	0.418^§^
Local (incl in transit)	24 (3.4)	10 (4.4)	
Regional lymph node	37 (5.3)	6 (2.6)	0.093
Distant	93 (13.4)	29 (12.7)	
Time to any relapse			
Median (IQR)	1.97 (1.17–3.48)	1.93 (1.04–3.09)	0.482
Anytime regional lymph node relapse in SLNB negative			
Regional lymph node	27 (5.0)	7 (3.7)	0.457
False negative rate	14.9	15.6	0.915

^a^Data available for 693 patients on the same day surgery group and 229 patients in next day surgery group

^b^Data available for 693 patients on the same day surgery group and 228 patients in next day surgery group

^c^Data available for 694 patients on the same day surgery group and 229 patients in the next day surgery group; values are number of cases (percentage) unless otherwise indicated

^‡^*χ*^2^ test

^§^*χ*^2^ test for trend

### Same day or next day SLNB after LSG does not influence outcomes

**Fig. 1:**
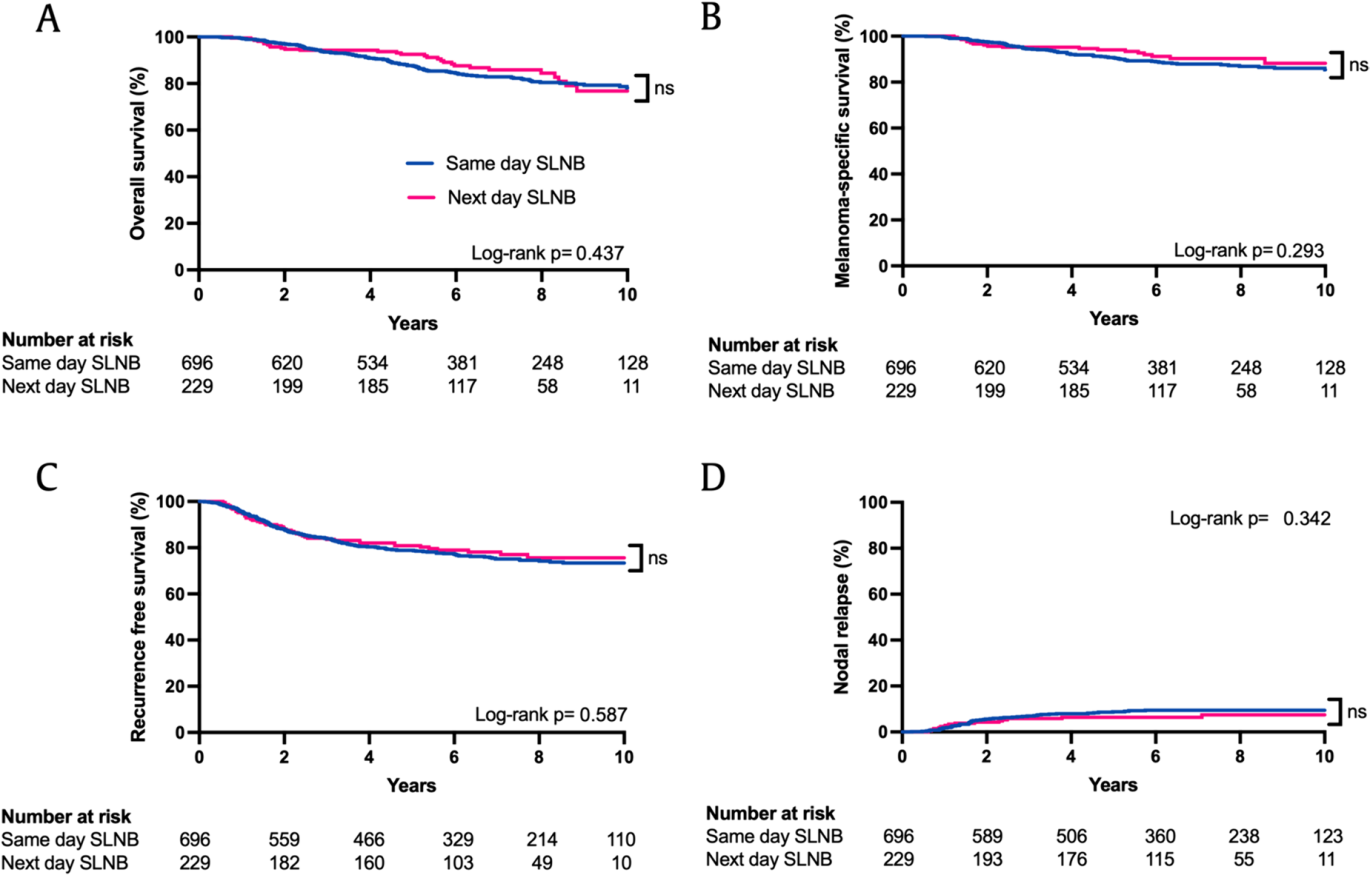
Kaplan–Meier survival estimates according to timing of SLNB after lymphoscintigraphy. **A** Overall survival *P* = 0.437, **B** melanoma-specific survival *P* = 0.293, **C** recurrence-free survival *P* = 0.587, **D** nodal relapse *P* = 0.342 (Log-rank test)

There was no significant difference in OS, MSS or PFS between patients who had same day or next day surgery (Fig. 1a–c). Additionally, there was no significant difference in the nodal recurrence pattern in both groups (Fig. 1d) or in distant recurrence (Supplementary Fig. 1). The time to relapse was not significantly different between the two groups (Table 2, median 1.97 years vs. 1.93 years, *P* = 0.482).

**Fig. 2:**
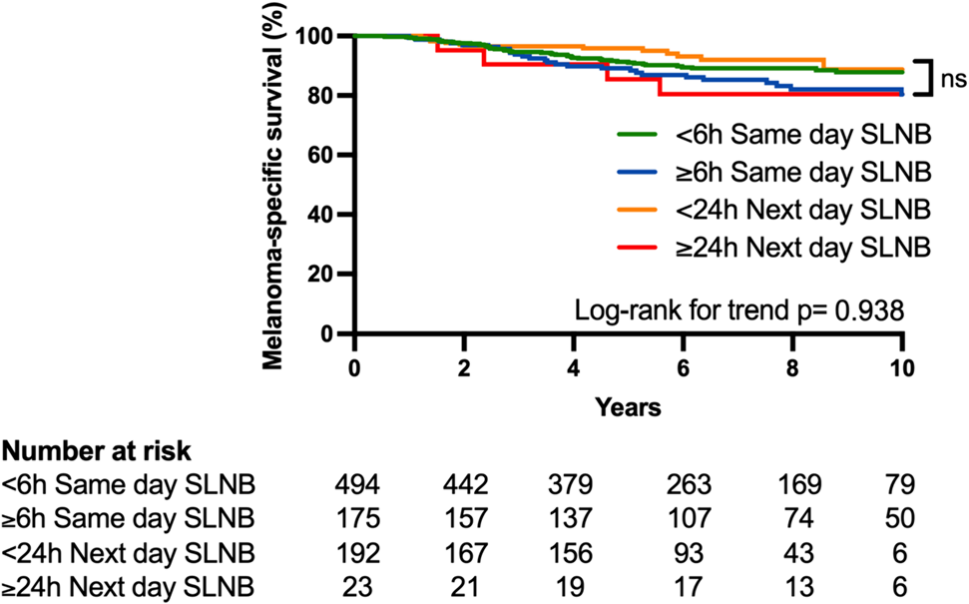
Kaplan–Meier Melanoma-specific survival estimate stratified in four groups by timing of SLNB after lymphoscintigraphy. *P* = 0.938 (Log-rank test for trend). Numerical time interval between LSG and SLNB not available for 41 patients

To further investigate the effect of time interval between tracer injection and SLNB on outcomes, we substratified the cohort in to 4 groups (Same day < 6 h, same day ≥ 6 h, next day < 24 h and next day ≥ 24 h). Data were available for 883 patients, with a range of 1.44–30.48 h (Supplementary Fig. 2). There were no observed differences in OS, MSS or PFS between the groups (Fig. 2 and Supplementary Fig. 3). Dividing the group alternatively in 5 cohorts did not demonstrate any survival differences (< 4 h, 4 to < 8 h, 8 to < 12 h, 12 to < 24 h, and ≥ 24 h; data not shown).

### Same day or next day SLNB after LSG is not an independent predictor of outcome

Cox multivariable analysis demonstrated age, Breslow thickness, and sentinel node status to be independent predictors of OS, MSS, and DFS in this cohort as expected and detailed in Table 3. Ulceration was an independent predictor for OS and PFS, but not for MSS (*P* = 0.047, < 0.001 and 0.199 respectively). Timing of surgery (same day vs next day) was not an independent predictor of OS, MSS or PFS (*P* = 0.149, *P* = 0.238, and *P* = 0.514 respectively).

**Table 3:** Multivariable Cox proportional hazards analysis of independent risk factors for overall, melanoma-specific and progression-free survival in combined dataset (*n* = 925)

	Overall survival		Melanoma-specific survival		Progression-free survival	
	Hazard ratio	*P*	Hazard ratio	*P*	Hazard ratio	*P*
Age	1.05 (1.03–1.07)	< 0.001	1.03 (1.02–1.05)	< 0.001	1.02 (1.01–1.03)	< 0.001
Breslow thickness	1.12 (1.05–1.17)	< 0.001	1.12 (1.05–1.18)	< 0.001	1.11 (1.07–1.15)	< 0.001
Ulceration (yes vs. no)	1.47 (1.00–2.14)	0.047	1.35 (0.85–2.13)	0.199	1.96 (1.43–2.69)	< 0.001
Gender (male vs. female)	1.43 (1.01–2.02)	0.043	1.26 (0.83–1.93)	0.285	1.15 (0.67–1.54)	0.328
Timing of Surgery (next day vs. same day)	0.74 (0.48–1.10)	0.149	0.73 (0.42–1.20)	0.238	0.89 (0.63–1.24)	0.514
Sentinel node status (positive vs. negative)	3.18 (2.22–4.54)	< 0.001	6.35 (4.07–10.01)	< 0.001	4.23 (3.13–5.73)	< 0.001

Values in parentheses are 95 per cent confidence intervals

## Discussion

Central to the utility of SLNB, is the accuracy of the preoperative LSG to identify the lymphatic drainage pattern from the skin. The first descriptions of the sentinel node technique in Europe for melanoma and breast cancer utilised ^99m^Tc-labelled nanocolloid, with the LSG done the day before surgery, with dual localisation with an intravital blue dye [13, 14].The choice of tracer for sentinel node localisation has evolved in parallel in different countries depending on local availability and regulations, with ^99m^Tc-labelled sulphur colloid, ^99m^Tc-labelled antimony sulphide colloid and ^99m^Tc-labelled nanocolloid forming the most used labels in North America, Australasia and Europe respectively. In addition, a ^99m^Tc-labelled CD206 receptor binding tracer (Tilmanocept) is licensed for use in the USA and Europe since 2013.

The timing of surgery after LSG also varies across different institutions, with both same day and next day schedules routinely used in Europe and Australasia, and same day scheduling alone more prevalent in the USA. Early reports investigating the concordance and identification rates of the sentinel node between the same day and next day scheduling were reported to have similar outcomes, and therefore the European Association for Nuclear Medicine (EANM) recommends that either scheduling is acceptable for both melanoma and breast cancer [3, 15–17].

A key recent and unexpected observation was made by O’Leary and colleagues [4], that there was a 10.7% overall survival benefit at 8 years after surgery when ^99m^Tc-labelled nanocolloid was used in the same day schedule as compared to a next day protocol. There was no difference in the lymph node recurrence rate in the two groups (5.2% vs. 4.6% respectively). This cohort was extended by combining outcomes from two additional centres [5] reporting a 4% overall survival benefit at 5 years, and no significant difference in the cumulative nodal relapse. This surprising finding was hypothesised to be due to incorrect sampling of the true SLN because of tracer migration in the next day SLNB group. However, the sentinel lymph node positivity rate was similar in both groups (16.3% vs. 15.1%) [5]. These two studies were the first reports of looking at the long-term outcome differences for adopting the same day or next day surgical scheduling.

A similar question was posed for melanoma outcomes using ^99m^Tc-labelled antimony sulphide colloid by Thompson and colleagues [6] investigating differences between the same day or next day surgery, and no survival outcome differences were noted. No outcome differences have yet been reported with other radiotracers.

Our present study does not show the same outcome differences in a comparable cohort of patients using ^99m^Tc-labelled nanocolloid. We do not observe overall survival, melanoma specific survival, progression free survival or nodal recurrence differences between the same day or next day SLNB after LSG with ^99m^Tc-labelled nanocolloid. Although our number of next day cases is smaller than the reports above, it is powered to be able to detect such a large overall survival benefit, especially given the longer median follow up of over 6 years. In the multicentre report from Moncrieff and colleagues [5], the three centres participating had different scheduling strategies, and significant differences in key indicators such as Breslow thickness, although other variables such as age and gender were matched. The nodal relapse as first site of recurrence and median follow up is not reported between the three sites.

We do not find significant differences in nodal relapse either as first site of recurrence, or at any time during follow up. Additionally, we do not find a difference in time to relapse. This is in contrast to the studies reported above, where nodal relapse was not significantly different between the same day and next day surgery groups as a cumulative incidence, but was significant if only the first site of relapse was considered [4, 5].

Tracer migration is a valid concern where the time interval between LSG and surgery is prolonged, and has been investigated in several studies in melanoma [6, 15, 18, 19] and breast cancer [14, 20–26] using ^99m^Tc-labelled colloid, sulfur colloid, antimony sulphide colloid and Tilamnocept. In studies which have performed LSG the day before surgery, and combined with a delayed scan without tracer re-injection the following day, the majority of scans showed no changes in uptake in the identified sentinel node, irrespective of tracer used [6, 15, 22]. In studies where the same day and next day protocols were evaluated by the identification rates of the sentinel lymph node without re-imaging, the majority of studies show no difference in number of lymph nodes removed and sentinel lymph node positivity rates providing indirect evidence of adequacy of sampling [21, 22]. The only study demonstrating a difference in the number of lymph nodes removed as sentinel on the same day vs. next day surgery scheduling is difficult to directly interpret [19]. In this study, Chakera and colleagues investigated two scheduling groups performed at different hospitals, with the same day surgery group receiving 13 MBq of ^99m^Tc-labelled nanocolloid, and the next day surgery group receiving 100 MBq with some patients being imaged on the same day, while others being imaged on the next day after radiotracer injection. Our present study did not demonstrate a significant difference in the number of sentinel lymph nodes removed from either same day or next day SLNB (1.0 vs. 1.0 median nodes, Table 2). It is important to highlight that it is standard practice to use a triple localisation technique in our institution using a Patent blue intraoperative injection providing further confidence regarding the accuracy of sentinel node identification.

Advantages of our current study include the long follow up period, and a single institution carrying out all procedures including head and neck melanomas. Limitations of our study include the retrospective nature of the cohort, no explicit randomisation, and fewer next day sentinel lymph node biopsies.

As ^99m^Tc-labelled nanocolloid is widely used in Europe by surgical oncologists for sentinel node identification in melanoma and breast cancer amongst others, it is important to consider the implications of the present study. We do not find identification or long-term outcome differences between same day or next day surgery, and would encourage other centres to examine their cohorts, and combine data where similar protocols exist, to build a wider body of evidence. Where operational capacity allows, same day scheduling LSG and SLNB scheduling is preferred, and has been our exclusive strategy from 2020. Where next day cannot be avoided, it is incumbent on surgeons to discuss the ambiguity in the outcomes and make use of alternative tracers such as ^99m^Tc-labelled Tilmanocept or additional day of surgery re-imaging to provide confidence in the correct identification of the sentinel lymph node.

## Conflict of interest

AR is supported by a Cancer Research UK/Royal College of Surgeons Clinician Scientist Fellowship (C64667/A27958) and has received honoraria from the Alliance for Cancer Early Detection, and the British Association for Plastic, Reconstructive and Aesthetic Surgery (all nonprofit organisations). JB and AJD report paid consultancy from Norgine BV (< 500,000 Yen). Other authors declare no conflicts of interest or financial support.

## Supplementary Information

Below is the link to the electronic supplementary material.

Supplementary file1 (PDF 499 KB)

## Data Availability

The data underlying this article will be shared on reasonable request to the corresponding author.

## References

[1] Morton DL, Thompson JF, Cochran AJ, Mozzillo N, Nieweg OE, Roses DF, et al. Final trial report of sentinel-node biopsy vs. nodal observation in melanoma. N Engl J Med. 2014;370(7):599–609. 24521106 10.1056/NEJMoa1310460 PMC4058881

[2] Sondak VK, Zager JS. Who is to blame for false-negative sentinel node biopsies in melanoma? Ann Surg Oncol. 2010;17(3):670–3. 19953329 10.1245/s10434-009-0857-y PMC2820671

[3] Bluemel C, Herrmann K, Giammarile F, Nieweg OE, Dubreuil J, Testori A, et al. EANM practice guidelines for lymphoscintigraphy and sentinel lymph node biopsy in melanoma. Eur J Nucl Med Mol Imaging. 2015;42(11):1750–66. 26205952 10.1007/s00259-015-3135-1

[4] O’Leary FM, Beadsmoore CJ, Pawaroo D, Skrypniuk J, Heaton MJ, Moncrieff MD. Survival outcomes and interval between lymphoscintigraphy and SLNB in cutaneous melanoma- findings of a large prospective cohort study. Eur J Surg Oncol. 2018;44(11):1768–72. 30343702 10.1016/j.ejso.2018.06.011

[5] Moncrieff MD, O’Leary FM, Beadsmoore CJ, Pawaroo D, Heaton MJ, Isaksson K, et al. Effect of delay between nuclear medicine scanning and sentinel node biopsy on outcome in patients with cutaneous melanoma. Br J Surg. 2020;107(6):669–76. 32077090 10.1002/bjs.11460

[6] Thompson JF, London K, Uren RF, Pennington TE, Saw RPM, Lo SN. Same-day or next day sentinel node biopsy after lymphoscintigraphy for melanoma using 99m Tc-labelled antimony sulphide colloid. Br J Surg. 2020;107(13):1773–9. 32820818 10.1002/bjs.11844

[7] Gershenwald JE, Scolyer RA, Hess KR, Thompson JF, Long GV, Ross MI, et al. Melanoma of the skin. In: Amin MB, editor., et al., AJCC cancer staging manual. 8th ed. Berlin: Springer International Publishing; 2017. p. 563–85.

[8] Pasha T, Arain Z, Buscombe J, Aloj L, Durrani A, Patel A, et al. Association of complex lymphatic drainage in head and neck cutaneous melanoma with sentinel lymph node biopsy outcomes. JAMA Otolaryngol-Head Neck Surg. 2023. 10.1001/jamaoto.2023.1585. 36892824 10.1001/jamaoto.2023.0076 PMC9999281

[9] Cook MG, Green MA, Anderson B, Eggermont AMM, Ruiter DJ, Spatz A, et al. The development of optimal pathological assessment of sentinel lymph nodes for melanoma. J Pathol. 2003;200(3):314–9. 12845627 10.1002/path.1365

[10] Marsden JR, Newton-Bishop JA, Burrows L, Cook M, Corrie PG, Cox NH, et al. Revised UK guidelines for the management of cutaneous melanoma 2010. Br J Dermatol. 2010;163(2):238–56. 20608932 10.1111/j.1365-2133.2010.09883.x

[11] National Institute for Health and Care Excellence. Melanoma : assessment and management (NG14). 2015. 26334080

[12] Faries MB, Thompson JF, Cochran AJ, Andtbacka RH, Mozzillo N, Zager JS, et al. Completion dissection or observation for sentinel-node metastasis in melanoma. N Engl J Med. 2017;376(23):2211–22. 28591523 10.1056/NEJMoa1613210 PMC5548388

[13] Kapteijn BAE, Nieweg OE, Liem I, Mooi WJ, Balm AJM, Muller SH, et al. Localizing the sentinel node in cutaneous melanoma: gamma probe detection vs. blue dye. Ann Surg Oncol. 1997;4(2):156–60. 9084853 10.1007/BF02303799

[14] Veronesi U, Paganelli G, Galimberti V, Viale G, Zurrida S, Bedoni M, et al. Sentinel-node biopsy to avoid axillary dissection in breast cancer with clinically negative lymph-nodes. Lancet. 1997;349(9069):1864–7. 9217757 10.1016/S0140-6736(97)01004-0

[15] Kalady MF, White DC, Fields RC, Coleman RE, Schuler FR, Seigler HF, et al. Validation of delayed sentinel lymph node mapping for melanoma. Cancer J. 2001;7(6):503–8. 11769863

[16] Chakera AH, Hesse B, Burak Z, Ballinger JR, Britten A, Caracò C, et al. EANM-EORTC general recommendations for sentinel node diagnostics in melanoma. Eur J Nucl Med Mol Imaging. 2009;36(10):1713–42. 19714329 10.1007/s00259-009-1228-4

[17] Giammarile F, Alazraki N, Aarsvold JN, Audisio RA, Glass E, Grant SF, et al. The EANM and SNMMI practice guideline for lymphoscintigraphy and sentinel node localization in breast cancer. Eur J Nucl Med Mol Imaging. 2013;40(12):1932–47. 24085499 10.1007/s00259-013-2544-2

[18] Oldan JD, James OG, Mosca PJ, Tyler DS, Borges-Neto S. Two-day lymphoscintigraphic imaging for melanoma. Nucl Med Commun. 2014;35(8):870–5. 24781011 10.1097/MNM.0000000000000136

[19] Chakera AH, Lock-Andersen J, Hesse U, Nürnberg BM, Juhl BR, Stokholm KH, et al. One-day or two-day procedure for sentinel node biopsy in melanoma? Eur J Nucl Med Mol Imaging. 2009;36(6):928–37. 19153733 10.1007/s00259-008-1036-2

[20] Pijpers R, Meijer S, Hoekstra OS, Collet GJ, Comans EFI, Boom RPA, et al. Impact of lymphoscintigraphy on sentinel node identification with technetium-99m-colloidal albumin in breast cancer. J Nucl Med. 1997;38(3):366–8. 9074519

[21] Van Esser S, Hobbelink M, Van Isselt JW, Mali WPTM, Borel Rinkes IHM, Van Hillegersberg R. Comparison of a 1-day and a 2-day protocol for lymphatic mapping and sentinel lymph node biopsy in patients with nonpalpable breast cancer. Eur J Nucl Med Mol Imaging. 2009;36(9):1383–7. 19319528 10.1007/s00259-009-1114-0 PMC2724640

[22] Taumberger N, Pernthaler B, Schwarz T, Bjelic-Radisic V, Pristauz G, Aigner RM, et al. Lymphoscintigraphy for sentinel lymph node biopsy in breast cancer: do we need a delayed image? Breast Care. 2020;15(1):55–9. 32231498 10.1159/000496504 PMC7098320

[23] Unkart JT, Proudfoot J, Wallace AM. Outcomes of “one-day” vs “two-day” injection protocols using Tc-99m tilmanocept for sentinel lymph node biopsy in breast cancer. Breast J. 2018;24(4):526–30. 29498443 10.1111/tbj.13002 PMC6043364

[24] Yeung HWD, Cody HS, Turlakow A, Riedel ER, Fey J, Gonen M, et al. Lymphosintigraphy and sentinel node localization in breast cancer patients: a comparison between 1-day and 2-day protocols. J Nucl Med. 2001;42(3):420–3. 11337517

[25] Mount MG, White NR, Nguyen CL, Orr RK, Hird RB. Evaluating one day vs. two days preoperative lymphoscintigraphy protocols for sentinel lymph node biopsy in breast cancer. Am Surg. 2015;81(5):454–7. 25975327

[26] Wang H, Heck K, Pruitt SK, Wong TZ, Scheri RP, Georgiade GS, et al. Impact of delayed lymphoscintigraphy for sentinel lymphnode biopsy for breast cancer. J Surg Oncol. 2015;111(8):931–4. 25953313 10.1002/jso.23915

