# Supplementary material for: Next day sentinel node biopsy for melanoma after lymphoscintigraphy using 99mTc-labelled nanocolloid does not adversely affect long-term outcomes
Source: Ann Nucl Med. 2024 Sep 16;39(1):77–85. doi: 10.1007/s12149-024-01980-y (PMC11706919; doi:10.1007/s12149-024-01980-y)
Supplement: Supplementary file 1 — Supplementary file1 (PDF 499 KB) [file 12149_2024_1980_MOESM1_ESM.pdf]

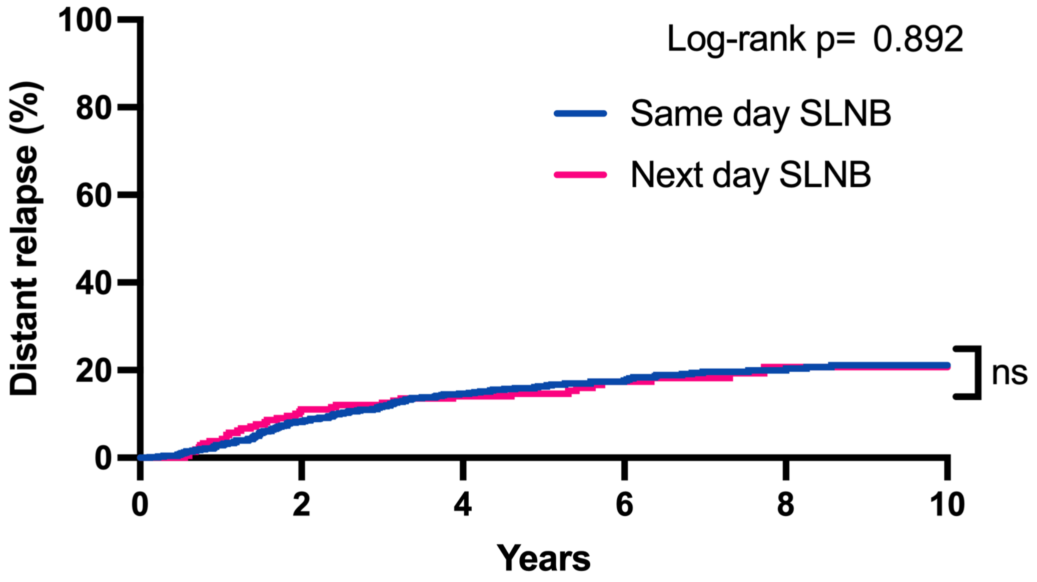

Number at risk

Same day SLNB	696	582	493	347	223	115
Next day SLNB	229	184	167	108	52	10

Supplementary Figure 1: Kaplan-Meier distant relapse estimates according to timing of SLNB after lymphoscintigraphy. *P*=0.892 (Log-rank test).

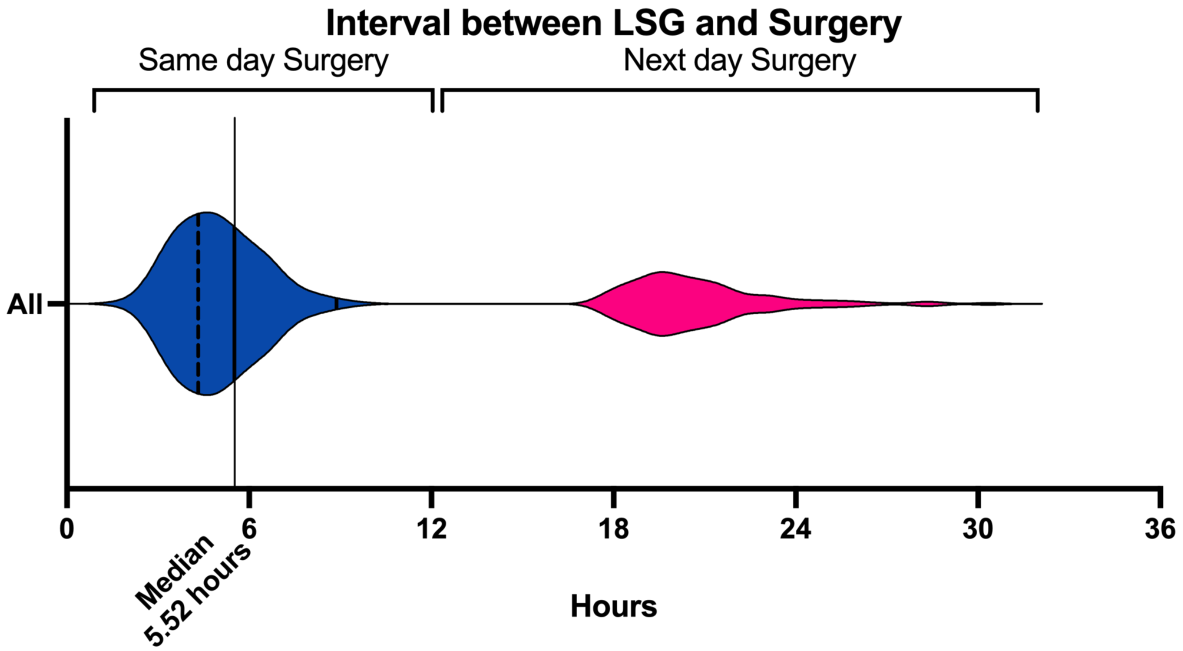

Supplementary Figure 2: Distribution of time interval between lymphoscintigraphy and sentinel node biopsy. N=884, data not available for 41 patients, Median=5.52 hours, IQR 4.32-8.88 hours, Min-Max 1.44-30.5 hours.

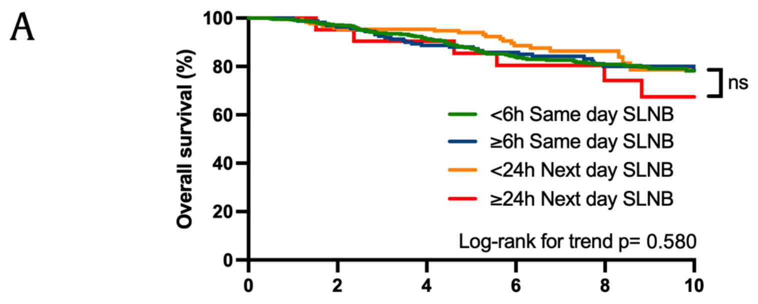

Number at risk	0	2	4	6	8	10
<6h Same day SLNB	494	442	379	263	169	79
≥6h Same day SLNB	175	157	137	107	74	50
<24h Next day SLNB	192	167	156	93	43	6
≥24h Next day SLNB	23	21	19	17	13	6

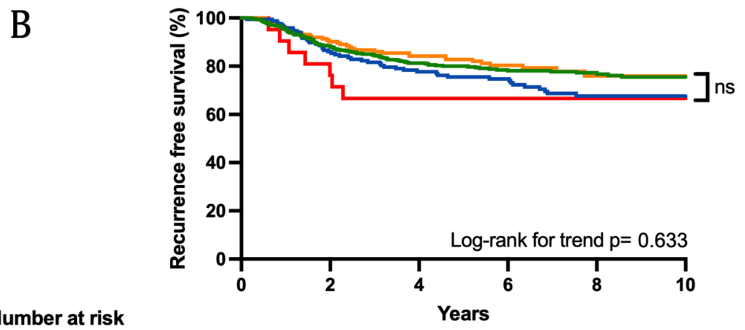

Number at risk	0	2	4	6	8	10
<6h Same day SLNB	494	400	334	228	147	69
≥6h Same day SLNB	175	139	117	92	62	42
<24h Next day SLNB	192	155	137	83	36	6
≥24h Next day SLNB	23	17	14	14	12	5

Supplementary Figure 3: Kaplan-Meier survival estimates stratified in four groups by timing of SLNB after lymphoscintigraphy. A Overall survival P=0.580, B Recurrence-free survival P=0.633 (Log-rank test for trend). Numerical time interval between LSG and SLNB not available for 41 patients.
